# Conversion of Germ Cells to Somatic Cell Types in *C. elegans*

**DOI:** 10.3390/jdb8040024

**Published:** 2020-10-07

**Authors:** Nida ul Fatima, Baris Tursun

**Affiliations:** 1 Berlin Institute of Medical Systems Biology, 10115 Berlin, Germany; 2 Max Delbrück Center for Molecular Medicine in the Helmholtz Association, 13125 Berlin, Germany

**Keywords:** germline, germ cell, reprogramming, *C. elegans*, epigenetics, chromatin

## Abstract

The potential of a cell to produce all types of differentiated cells in an organism is termed totipotency. Totipotency is an essential property of germ cells, which constitute the germline and pass on the parental genetic material to the progeny. The potential of germ cells to give rise to a whole organism has been the subject of intense research for decades and remains important in order to better understand the molecular mechanisms underlying totipotency. A better understanding of the principles of totipotency in germ cells could also help to generate this potential in somatic cell lineages. Strategies such as transcription factor-mediated reprogramming of differentiated cells to stem cell-like states could benefit from this knowledge. Ensuring pluripotency or even totipotency of reprogrammed stem cells are critical improvements for future regenerative medicine applications. The *C. elegans* germline provides a unique possibility to study molecular mechanisms that maintain totipotency and the germ cell fate with its unique property of giving rise to meiotic cells Studies that focused on these aspects led to the identification of prominent chromatin-repressing factors such as the *C. elegans* members of the Polycomb Repressive Complex 2 (PRC2). In this review, we summarize different factors that were recently identified, which use molecular mechanisms such as control of protein translation or chromatin repression to ensure maintenance of totipotency and the germline fate. Additionally, we focus on recently identified factors involved in preventing transcription-factor-mediated conversion of germ cells to somatic lineages. These so-called reprogramming barriers have been shown in some instances to be conserved with regard to their function as a cell fate safeguarding factor in mammals. Overall, continued studies assessing the different aspects of molecular pathways involved in maintaining the germ cell fate in *C. elegans* may provide more insight into cell fate safeguarding mechanisms also in other species.

## 1. Loss of Germline Totipotency and Teratoma Formation

### 1.1. Translational Regulators Maintain Germline Totipotency and Prevent Teratomas

The totipotency of the germline is maintained by multiple levels of control. In the absence of these mechanisms, germ cells can differentiate to somatic cells thereby forming germline teratomas with co-occurrence of multiple somatic lineages at once. Such teratomas form upon loss of the translational regulators GLD-1 (ortholog of human KH-domain containing RNA-binding protein QKI) and MEX-3 (ortholog of human MEX3A RNA-binding protein) and germ cells acquire somatic fates including muscle, neuron and intestine [1]. These teratomas arise spontaneously in most of the *gld-1 mex-3* double mutants, and in *gld-1* mutant alone to a lesser extent. Strikingly, induction of the somatic fates is accompanied by acquisition of cell type-specific characteristics. These include filaments and adhesive structures observed in cells acquiring muscle fate or birefringent, auto-fluorescent granules typical for intestinal cells indicating that germ cells converted to somatic lineages. This is further evidenced by the expression of the pan-neuronal fate reporter *unc-119::GFP* in the germline teratomas.

In *gld-1 mex-3* double mutants, central regions of the germ line show a marked reduction in size and number of the germ cell-specific P granules, which is likely to be hallmark of precursors that undergo transdifferentiation to somatic fates. This central region of the germ line consists of meiotic germ cells, and entry into meiosis seems to be critical for the somatic fate induction. Interestingly, subsequent findings in a later study by Updike et al. (described below) provide evidence that loss of P granules may be a cause of germline differentiation to somatic lineages [2].

Consistent with the observed muscle fate in germline teratomas of *gld-1 mex-3* double mutants, the myogenic basic helix-loop-helix (bHLH) transcription factor HLH-1 [3] was detected in large numbers of the germline nuclei [1]. Depletion of its upstream caudal-type homeodomain transcriptional regulator PAL-1 diminished HLH-1-positive nuclei as well as the number of muscle-like cells in germline teratomas. Although PAL-1 appears to be involved in the ectopic acquisition of muscle fate in the germline, it is unlikely that its inappropriate expression alone is sufficient to induce transdifferentiation [1]. Rather, expression of transcription factors such as PAL-1 and HLH-1 in combination with affected maintenance of the germ cell fate may allow conversions to somatic cell types. These defects could include loss of P granules as observed in the *gld-1 mex-3* mutants and also defective chromatin regulation, which altogether lead to teratoma formation in the germline.

### 1.2. P Granules Safeguard Germline Identity

Another important safeguard of germline totipotency and germ cell fate is specialized ribonucleoprotein structures termed P granules that are also known as *C. elegans* germline granules [4]. These perinuclear RNA granules are highly specific to the germline and are composed of mainly two classes of RNA-binding proteins, which belong to RGG domain-containing proteins: PGL-1 and PGL-3; and GLH-1–4 DEAD box proteins, which can have RNA helicase activity [4]. While they are characteristic of germ cells and are known to be required for fertility, their potential role in the maintenance of germ cell identity was revealed by Updike et al. [2]. Upon simultaneous depletion of the two P granule proteins PGL-1 and PGL-3 together with GLH-1 and GLH-4, which are required for P granule localization to the nuclear periphery (*glh-1 and glh-4*), expression of the body wall muscle myosin MYO-3 [5], and the pan-neuronal gene reporters *unc-119::GFP* as well as *unc-33:GFP* could be detected in the germline. Additionally, the GFP-positive germ cells exhibited projections as seen in the germline teratomas of *mex-3; gld-1* double mutants [2]. Yet, no expression of neuronal markers that report terminally differentiated neurons could be observed, raising the possibility that transdifferentiation of germ cells to neurons is incomplete upon depletion of P granules. However, by inducing ectopic expression of the Zn-finger transcription factor CHE-1, which is promoting the terminal differentiation of neurons to the glutamatergic taste neuron identity (termed ASE) [6,7], germ cells lacking PGL-1, PGL-3, GLH-1, and GLH-4 started expression of a terminal ASE neuron fate marker.

Notably, the phenotype of the neuron-like cells generated upon loss of P granules appears to be similar to that obtained in germline teratomas of *mex-3, gld-1* double mutants. However, the overall phenomenon appears to be distinct in terms of mitotic versus meiotic germ cells undergoing reprogramming in P granule-depleted animals. Teratoma formation in *mex-3, gld-1* double mutants require entry into meiosis, which is not the case for germ cell conversion to somatic cell in the P granule-depleted animals, where mitotic germ cells gave rise to somatic cell types [1,2].

Overall, P granules act as a post transcriptional barrier for differentiation of germ cell to somatic cell types thereby maintaining the totipotency of germ cells.

### 1.3. Epigenetic Regulators SPR-5/LSD1 and LET-418/Mi2 Protect Germline Fate

Besides the protection of germline totipotency by translational regulators such as GLD-1 and MEX-3, epigenetics-based safeguarding of the germ identity is also crucial to maintain the germline and germ cell fate. The evolutionary conserved chromatin regulators SPR-5 (worm homolog of Lysine-specific histone demethylase LSD-1) and LET-418 (member of chromatin remodeling nucleosome remodeling and deacetylase complex NurD and MEP1) have been identified to be required for safeguarding the germline [8]. SPR-5 and LET-418-containing complexes are involved in development and maintenance of germline stem cells. Double mutants of *spr-5* and *let-418* are sterile and exhibit abnormal oval shaped gonads. The germ cells lose their characteristic P granules and replicate actively with mitosis occurring in the entire gonad instead of undergoing meiosis. Moreover, in *spr-5(by134)* and *let-418(RNAi)* mutants, the germ cells acquire teratoma-like characteristics and activate somatic differentiation programs that lead to expression of pan-neuronal genes including *unc-119* (muscle-expressed lipid-binding protein)*, rab-3* (ortholog of human RAB3A, neuron-specific GTPase) and *unc-33* (homolog of CRMP). Correspondingly, teratoma cells also display morphological similarities to neurons. In addition to the neuronal fate, also GFP reporter expression of the muscle proteins UNC-97 (zinc finger domain containing muscle-specific protein) and MYO-3 (Myosin) could be observed, however, predominantly in germlines of old adults [8].

Mechanistically, the absence of the demethylase SPR-5 allows elevated levels of H3K4 methylation, which suggests increased chromatin activation [8]. The highly conserved methyltransferase “Complex of Proteins Associated with Set1” (COMPASS) [9] mediated the increase in H3K4 methylation, which appears to underlie the spontaneous germ cell reprogramming to somatic fates in the absence of SPR-5 and the chromatin remodeler LET-418. Interestingly, LET-418 had been shown previously to play a role in preventing the expression of germline-specific genes in soma to ensure proper differentiation of somatic lineages [10]. Hence, it is possible that LET-418 is a general safeguard of cellular identities in a context-dependent manner.

Together, the finding that SPR-5 and LET-418 safeguard the germline identity indicates that the perturbation of epigenetic regulation in the germline results in disruptive cell fate control during germ cell development.

### 1.4. Epigenetic Regulators SET-2/SETD1A and WDR-5.1/WDR5 Maintain Germ Cell Pluripotency

Methylation of Histone H3 at Lys 4 (H3K4me) is catalyzed by the conserved SET1/MLL methyltransferase complex. SET-2/SET1D1A and WDR-5.1/WDR5 are part of this complex and are required for H3K4 methylation in the germline [11,12,13]. It was shown that SET-2 and WDR-5.1 promote the expression of genes related to germline function and share a common role in repression, of somatic gene expression in the germline [12]. Consequently, mutants have significantly decreased levels of H3K4 methylation and they exhibit temperature-sensitive progression of sterility [11,12,13]. Notably, at the onset of sterility in the *set-2* mutants the neuronal *unc-119::GFP* reporter expression is observed in the germline [12]. Furthermore, quantitative PCR of *set-2* mutants revealed expression of somatic genes including *ceh-2, ceh-20*, and *ceh-43*, that are involved in differentiation of neurons [12]. Their expression increased over generations in the germ lines of the mutant animals. However, expression of the *haf-9* gene, which is related to the intestinal fate, or the master transcription factor of the intestinal fate ELT-2 (GATA type) could not be detected. Yet, muscle marker genes such as *unc-120* (encoding a MADS-box transcription factor) and genes encoding for muscle myosin could be detected in the germlines of *set-2* mutants, albeit with less occurrence than neuronal genes expression, suggesting that loss of *set-2* facilities primarily the conversion of germ cells into neuronal cells.

Interestingly, while such transdifferentiation of germ cells into somatic cells occurred also in *wdr-5.1* mutants, this phenomenon was not observed in mutants of *ash-2*, which encodes for subunit of the MLL methyltransferase complex thereby also contributing to H3K4 methylation [12]. The authors of the study speculate that in *ash-2* mutants, the germline-to-soma conversion is absent because expression levels of somatic genes are not sufficient to induce transdifferentiation despite the loss of H3K4 methylation as well as decreased expression of germline genes. In contrast, *set-2* and *wdr-5.1* mutants displayed a higher degree of somatic gene expression derepression in the germlines.

Interestingly, in addition to the decreased H3K4 methylation levels, the *set-2* mutants display altered levels of the repressive H3K9me3 and H3K27me3 chromatin marks. Astonishingly, H3K9me3 levels decreased while H3K27me3 levels increased indicating a highly disorganization of germline chromatin in these mutants [12]. Furthermore, the authors tested mutants of *hrde-1*, a component of the germline nuclear RNAi pathway [14], as they were described previously to have similar germline defects as *set-2* and *wdr-5.1* mutants [12]. Interestingly, *hrde-1* mutants also displayed changes in H3K9me3 and H3K27me3 levels as well as expression of the neuronal marker *unc-119::GFP* in the germline [12]. However, enhancement of somatic genes expression upon *hrde-1* co-depletion with *set-2* and *wrd-5.1* mutants suggest that HRDE-1 rather acts in pathways parallel to SET-2/WDR-5.1 involving, at least partially, repression of common gene targets.

**Figure 1:**
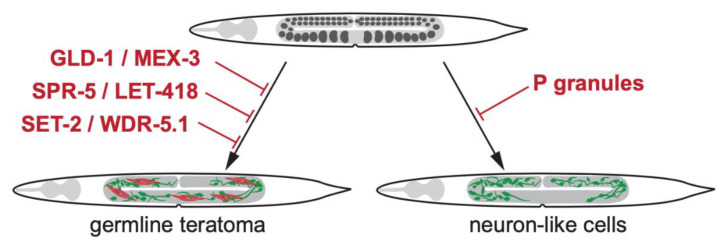
Loss of germline totipotency and teratoma formation. Upon loss of the translational regulators GLD-1 and MEX-3 germ cells differentiate to somatic cells thereby forming germline teratomas with co-occurrence of multiple somatic lineages at once including muscle, neuron and intestine [1]. Loss of the chromatin regulators SPR-5/LET-418 [8] and SET-2/WDR-5.1 [12] also result in germline teratomas. Loss of germline P granules leads to differentiation of germ cells to neuron-like cells, which do no acquire neuronal sub type identities [2].

Notably, overall levels of H3K4 methylation seem to be critical for maintaining the germline fate. Both, increased levels upon loss of SPR-5 and LET-418 [8], as well as decreased levels in *set-2* mutants cause loss of germline totipotency [12]. It is possible that increased H3K4 levels lead to ectopic activation of chromatin thereby allowing somatic gene expression. In contrast, decreased H3K4 levels may be insufficient to maintain a germline-specific gene expression pattern, which prevents ectopic expression of somatic genes. Nevertheless, the exact role of H3K4 methylation for maintaining the germline will require further investigation at the molecular level by assessing the germline-specific distribution in the genome and consequences on gene expression. Yet, these findings indicate that loss of epigenetic regulators (Figure 1) accompanied by altered chromatin regulation affect the epigenetic landscape in the germline and provides a context that is permissive for germ cell transdifferentiation into somatic cell types.

## 2. Transcription Factor-Induced Germ Cell Reprogramming to Somatic Fates

### 2.1. Loss of Histone Chaperone LIN-53 Permits Germ Cell Reprogramming by Transcription Factors

Cell fate conversion by the overexpression of specific transcription factors (TFs) which can induce reprogramming, is limited due to cellular maintenance and protection. During development, mechanisms arise that safeguard cell fates of somatic as well as germ cells to prevent perturbations of their states and identities. These safeguarding mechanisms often rely on epigenetic regulation as described above. Hence, forced expression of TFs that can induce ectopic fates in highly plastic cells such as developing cells during embryonic development, usually fail to induce conversion of germ cells to somatic identities [7,15].

In this context, the histone chaperone LIN-53 [16] has been identified to prevent direct reprogramming of germ cells into gustatory neurons (known as ASE) by the Zn-finger TF CHE-1 [7]. While overexpression of CHE-1 in embryos resulted in ectopic expression of the ASE neuron fate marker *gcy-5::GFP* in most embryonic cells, broad CHE-1 mis-expression in adults generally fails to do so in the soma and the germline. However, depletion of *lin-53* by RNAi and overexpression of CHE-1 in adults induced conversion of mitotic germ cells into neuron-like cells. The converted germ cells expressed markers for pan-neural fate (including *rab-3, snb-1, unc-119, unc-33, rgef-1*, SNB-1, UNC-10) as well as for specific neuron types (*gcy-5, ceh-36*). Importantly, germ cells lost their characteristic P-granules and PGL-1 expression accompanied by changes in cellular morphology. Converted germ cells lost their typical morphology in the mitotic zone of the germline and started to show nuclei and axo-dendritic projections overall, resembling neuronal cells [7].

In addition to the ASE neuron fate, *lin-53* knockdown allowed conversion of germ cells into either cholinergic or GABAergic motor neurons upon overexpression of the EBF-like TF UNC-3 or the Pitx-type homeodomain TF UNC-30, respectively [7]. Notably, converted germ cells display neuronal identity markers that are specific for the induced fate by the respective TF that is being overexpressed in the germline. For instance, germ cells converted to the glutamatergic ASE neuron fate by CHE-1 do not show marker expression of other neuron sub-types such as GABAergic and cholinergic identities. Hence, this conversion is distinct from the formation of teratomas in the germline resulting from the loss of the translational regulators such GLD-1 [1]. While germline teratomas result from undirected differentiation of germ cells to multiple somatic lineages, defined neuronal identities are obtained by using the above-mentioned TFs [7]. Furthermore, *lin-53* depletion alone without TF overexpression does not induce any detectable conversion or differentiation of germ cells. Rather, loss of LIN-53 creates permissiveness for germ cell reprogramming but not spontaneous differentiation to somatic lineages as seen upon depletion of other epigenetic regulators such as SPR-5, LET-418, and SET-2 [8,12].

Interestingly, while mitotic germ cells were shown to be undergoing reprogramming, mitosis or the cell cycle itself were not required for converting germ cells to neurons. Blocking the cell cycle by hydroxy urea or genetically by using a mutant background for the cell cycle regulator *emb-30* still allowed efficient germ cell reprogramming in the context of *lin-53* depletion and TF overexpression [17]. Yet, although *lin-53* was depleted by RNAi systemically in the entire adult body, the TF-induced conversion occurs in the germline only, which suggested a rather germline-specific mechanism of cellular safeguarding by the histone chaperone LIN-53.

### 2.2. LIN-53 Cooperates with PRC2 to Prevent Permits Germ Cell Reprogramming by TFs

Further studies on LIN-53′s role in protecting cellular identity revealed that it is functioning together with the Poly Comb Repressive Complex (PRC2) to safeguard germ cells from being reprogrammed by TFs [17]. PRC2 is a highly conserved epigenetic regulator, which represses chromatin by depositing H3K27 di- and tri-methyl marks (H3K27me2/3) in the germline of adult worms [18,19,20]. Depletion of PRC2 components (MES-2/Ezh2; MES-3; MES-6/Eed) leads to a loss of chromatin repression and to permissiveness for germ cell reprogramming to neuron and muscle-like cells upon overexpression of CHE-1 or the myogenic bHLH TF HLH-1 [17]. This germ cell reprogramming phenocopies *lin-53* depletion and the fact that *lin-53* RNAi results in global loss of H3K27me2/3 in the germline suggested that LIN-53 and PRC2 cooperate in safeguarding the germ cell fate.

Interestingly, also improper redistribution of H3K27me2/3 in germline chromatin, caused by the lack of the histone H3K36 methylating SET-domain protein MES-4 (H3K36 methylase), resulted in permissiveness for TF-induced cellular conversion [21]. Moreover, it was shown that sperm chromosomes lacking H3K27me3 inherit de-silenced chromatin to their offspring and that this de-repressed state is maintained due to antagonistic H3K36me3 modifications by MES-4 [22]. Consequently, inherited sperm chromosomes, which lack H3K27me3, caused germline chromatin de-repression and sterility in the progeny. Consistently, the de-repressed germline chromatin resulted in permissiveness for germ cell conversion to neurons as evidenced by expression of the pan-neuronal reporter *unc-119::GFP* and immunostaining for the neuronal SNARE protein UNC-64, which is involved in synaptic vesicle fusion ([22]. This was accompanied by the loss of the germ cell-specific component of germ granules PGL-1 and the component of the synaptonemal complex HTP-3. Overall, these results demonstrated that chromatin de-repression due to inherited H3K27me3-lacking sperm chromatin causes a transition towards a neuronal gene expression program in germ cells.

### 2.3. LIN-53 and PRC2 Antagonize Notch Signaling-Enhanced Germ Cell Reprogramming

**Figure 2:**
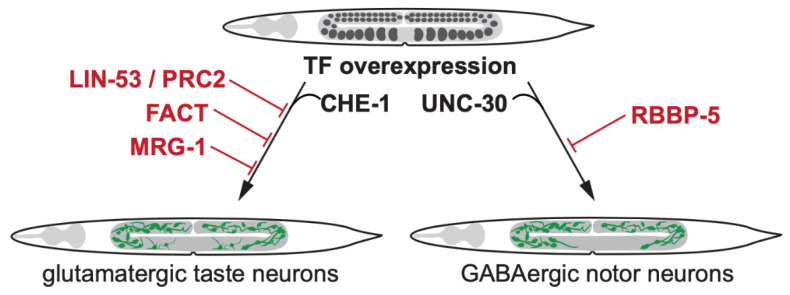
Transcription factor-induced germ cell reprogramming to somatic fates. Loss of the histone chaperone LIN-53 allows direct reprogramming of germ cells into glutamatergic taste neurons (known as ASE) by the Zn-finger TF CHE-1 [7]. LIN-53 cooperates with the Poly Comb Repressive Complex 2 (PRC2) to safeguard germ cells from being reprogrammed by TFs [17]. The heterodimeric histone chaperone FACT [28] and the chromodomain protein MRG-1 block germ cell conversion to glutamatergic neurons [35]. The Set1/MLL methyltransferase complex member RBBP-5 blocks UNC-30 TF-induced germ cell reprogramming to GABAergic neurons [40].

Further investigation of germ cell fate safeguarding by LIN-53 and PRC2 revealed that conversion to neurons is strongly enhanced in animals with increased germline Notch signaling [23]. The Notch receptor GLP-1 maintains the germ stem cell niche and mutants with a gain-of-function mutation for *glp-1(gof)* have elevated Notch signaling in their germline [24]. In combination with *lin-53* RNAi, *glp-1(gof)* mutants show a striking enhancement of germ cell reprogramming to neurons upon TF overexpression [23]. Transcriptome analysis of dissected gonads revealed that elevated Notch signaling activates PRC2-silenced genes, including expression of the H3K27 demethylase UTX-1. The transcriptional activation of *utx-1* is mediated by Notch-dependent transcription factor LAG-1. As UTX-1 is an antagonist of PRC2-mediated chromatin repression, loss of silenced chromatin results in the enhanced reprogramming of germ cells to neurons. Overall, H3K27me3-mediated chromatin silencing by PRC2 in cooperation with LIN-53 defines an epigenetic state that prevents TF-induced conversion of germ cells to somatic identities (Figure 2).

### 2.4. The Heterodimeric Histone Chaperone FACT Prevents TF-Induced Germ Cell Reprogramming

The histone chaperone FACT (facilitates chromatin transcription) is a heterodimeric complex and was previously known primarily for its role in promoting expression [25]. As a histone chaperone it disassembles nucleosomes to support RNA polymerase II during transcription [26].

By performing a whole-genome RNAi screen and using overexpression of the neuron fate-inducing TF CHE-1, as described earlier [7,27], subunits of the *C. elegans* FACT complex were identified to block germ cell conversion [28]. RNAi-mediated depletion of the FACT complex member HMG-3 allows germ cell-to-neuron conversion in *C. elegans*. Acquisition of neuronal morphology such as axo-dendritic projections was accompanied by pan-neuronal gene expression as well as expression of subtype-specific neuronal genes. Expression of neuronal genes upon germ cell reprogramming by CHE-1 was not only assessed based on transgenic reporter expression but also by applying single-molecule fluorescence in situ hybridization (smFISH) to confirm endogenous gene expression of neuronal genes.

Interestingly, it turned out that FACT has two isoforms in *C. elegans*. HMG-3 is exclusively expressed in the germline and therefore forms a germline-specific FACT complex with the ubiquitously expressed subunit SPT-16. The HMG-3 paralog HMG-4 (approximately 90% amino acid similarity with HMG-3) is predominantly expressed in the soma and thereby forms a somatic isoform of FACT together with SPT-16. Notably, also the somatic FACT is involved in cellular safeguarding by blocking the ectopic induction of neuronal fates in the intestine [28]. Although intestinal conversion to neuron-like cells did not cause obvious morphological changes, probably due to structural constraints, smFISH revealed that several neuron-specific genes were expressed.

Notably, depletion of FACT indicated an impairment of cell fate maintenance in the germline and the intestine, respectively. Expression of germ cell-specific markers including P granules and expression of the *pie-1* gene decreased upon RNAi against *hmg-3* without inducing CHE-1 TF overexpression [28]. A similar effect in intestinal cells was obtained by depletion of HMG-4, which led to decreased expression of intestinal fate marker genes including *elt-2*, *elt-7*, and *ges-1*, as well as loss of the gut-specific protein IFB-2 [28]. Assessment of chromatin accessibility by the “Assay for Transposase-Accessible Chromatin using sequencing” (ATAC-seq) [29] further confirmed loss of cell fate maintenance in these tissues upon RNAi against FACT subunits. Overall, this observation suggests that FACT safeguards germ cells and intestinal cells from being reprogrammed by maintaining their respective cellular identities.

Notably, FACT is highly conserved across worms and humans and the same study demonstrated that its loss in human cells also creates increased permissiveness for cellular reprogramming [28]. Depletion of the human FACT homologs using siRNAs (SSRP1 and SUPT16H) improved the reprogramming efficiency of human fibroblasts to induced pluripotent stem cells (iPSCs) and to neurons upon overexpression of previously described reprogramming-inducing TFs [28]. Chromatin and transcriptome analysis using ATAC-seq and RNA-seq, respectively, revealed that FACT affects chromatin accessibility and expression in both positive and negative manner. Interestingly, the increased expression observed upon depletion of FACT includes pluripotency promoting factors such as SAL4, while decreased expression of genes encoding for reprogramming inhibitors were also observed.

Overall, FACT acts as an evolutionarily conserved cellular reprogramming barrier by maintaining appropriate gene expression profiles to safeguard cell fates.

### 2.5. The Chromodomain Protein MRG-1 Counteracts TF-Mediated Germ Cell Reprogramming

MRG-1 is a worm ortholog of human MORF-related gene on chromosome 15 known as MRG15 [30,31]. The chromodomain protein MRG-1 is part of the NuA4 histone acetyltransferase complex [32] and was previously shown to be involved in a variety of roles during germline development such as progression to meiosis [33,34]. MRG-1 was recently identified as a novel factor in safeguarding germ cell identity against TF induced conversion. Depletion of *mrg-1* by RNAi with ectopic CHE-1 induction allows conversion of the germ cells to neurons displaying morphological and molecular characteristics typical of the acquired cell type [35]. As described before for LIN-53 and FACT, converted germ cells lose the germ cell-specific PIE-1 expression and P granules while expression of neuronal genes was acquired based on transgenic reporter expression as well as by smFISH confirming endogenous gene expression of neuronal genes [35].

Although the observed germ cell reprogramming phenotype upon loss of *mrg-1* is similar to those observed upon depletion of LIN-53 or PRC2, no change in the levels of LIN-53 or H3K27me2/3 was observed as it was shown in LIN-53 or PRC2-depleted germlines [17,35] (Figure 2). Thus, MRG-1 seems to act in a mechanism distinct from LIN-53 for the protection of germ cell identity. Indeed, soma- and germline-specific ChIP-seq analysis of genomic MRG-1 distribution identified that it predominantly binds loci carrying the active chromatin mark H3K36me3 [35]. This finding suggests that MRG-1 may protect the cellular identity by maintaining expression of germline components. Furthermore, immunoprecipitation coupled to mass spectrometry of endogenous MRG-1 proteins revealed novel interaction partners of MRG-1. Among the candidate interacting proteins was the SET domain protein SET-26 that has H3K9 methylation activity; and the *N*-acetylglucosamine (*O*-GlcNAc) transferase OGT-1 that has been proposed to be part of histone acetyltransferase-containing protein complexes [36,37,38]. Notably, *set-26* and *ogt-1* mutants showed an increase in the number of converted germ cells upon *mrg-1* RNAi indicating that these proteins may be relevant to the role of MRG-1 in protecting germ cell fate [35]. Furthermore, increase in H3K14ac in the *mrg-1* RNAi germline has been observed, which could result in decreased efficiency of H3K9 methylation [39]. Together, these effects may result in disruptive or redistributive effects on the overall chromatin signature, which possibly underlie the effects of decreased germ cell fate maintenance upon *mrg-1* depletion.

### 2.6. The Methyltransferase Complex Member RBBP-5 Blocks TF-Mediated Germ Cell Conversion

The Set1/MLL methyltransferase complex member RBBP-5 [13,32] was recently identified as another novel germ cell reprogramming barrier. It was identified in combination with LIN-53 depletion while screening for increased efficiency of germ cell reprogramming to GABAergic neurons [40]. While it was shown that depletion of LIN-53 also allows reprogramming of germ cells to GABAergic neurons upon overexpression of the Pitx-type homeodomain TF UNC-30 [7], the conversion rate is rather low when compared to germ cell reprogramming to ASE neuron-like cells by overexpressing CHE-1 [40]. To test whether germ cell reprogramming to GABAergic neurons could be enhanced upon co-depletion of other chromatin regulators with LIN-53, a novel double RNAi pipeline was applied which led to the identification of RBBP-5 as a novel germ cell reprogramming barrier.

The mechanism by which RBBP-5 safeguards the germ cell fate remains to be determined and could rely on its function in maintaining H3K4 methylation in the germline [11].

## 3. Outlook

Investigating the *C. elegans* germline is powerful in order to identify cell fate safeguarding mechanisms as summarized above. Moreover, most (if not all) of the identified safeguarding factors described in this review are highly conserved in mammals (Table 1) suggesting evolutionary conservation of mechanisms that maintain the germ cell fate. Yet, the maintenance and safeguarding function of these factors could be extended to somatic cells in mammals. For instance, while the histone chaperone LIN-53 protects the germline against TF-induced reprogramming in *C. elegans*, it was shown that the CAF-1 histone chaperone complex blocks TF-induced reprogramming of mouse embryonic fibroblasts to neurons and iPSCs [41]. The mammalian homolog of LIN-53, known as RBBP4/7 or CAF-1p48, is a core subunit of CAF-1, which reflects an analogous role of this conserved histone chaperone in counteracting TF-induced reprogramming. In addition, the identification of the heterodimeric histone chaperone FACT as a reprogramming barrier in *C. elegans* germline and soma as well as in human fibroblasts [28] further reflects a remarkable conservation of safeguarding factors during evolution. Notably, the whole-genome RNAi screen by which FACT was identified revealed a number of additional factors that antagonize TF-induced reprogramming of germ cells, which belong to different types of biological functions such as proteostasis, protein transport systems and even mitochondria [28]. Future studies will reveal the molecular pathways through which these factors safeguard the germline and whether their role in blocking TF-induced reprogramming is conserved in mammalian cells. A number of technological advancements provide the opportunity to dissect the exact molecular mechanisms of how the identified factors ensure totipotency and protect the germ cell fate researchers have been safeguarding in *C. elegans*. Technologies for transcriptome as well as chromatin accessibility analysis with single cell resolution (scRNA-Seq and scATAC-Seq) are available for other model systems and tissue types. Adopting these techniques for the *C. elegans* germline will be essential to improve our knowledge since most available data are based on dissected gonads containing a rather heterogeneous cell population of different states such as mitotic and meiotic germ cells.

Future work utilizing the power of genetics in *C. elegans* in combination with sophisticated molecular applications will advance our understanding of germline totipotency and safeguarding of the germ cell fate. Such knowledge has great potential to also improve techniques for generating new tissues by reprogramming approaches thereby providing new avenues for future regenerative medicine applications.

## Figures and Tables

**Table 1:** Overview of Safeguarding Factors.

	Loss of Germline Totipotency and Teratoma Formation		
Factor	Function	Human Homolog	Reference
GLD-1	Translational regulation	QKI	[1] Ciosk et al., 2006
MEX-3	Translational regulation	MEX3A	[1] Ciosk et al., 2006
SPR-5	Chromatin regulation:	LSD-1	[8] Käser-Pébernard et al., 2014
	Histone demethylation		
LET-418	Chromatin regulation:	CHD3	[8] Käser-Pébernard et al., 2014
	Nucleosome remodeling,		
	Histone deacetylation		
SET-2	Chromatin regulation:	SETD1A	[12] Robert et al., 2014
	H3K4 methylation		
WDR-5.1	Chromatin regulation:	WDR5	[12] Robert et al., 2014
	H3K4 methylation		
HRDE-1	RNA interference	-	[12] Robert et al., 2014
P granule proteins	RNA helicase activity	DDX4	[2] Updike et al., 2014
(PGL-1/-3, GLH-1-4)			
	**Antagonizing TF-Induced Germ Cell Reprogramming to Somatic Fates**		
LIN-53	Histone chaperone	RBBP4/7	[7] Tursun et al., 2011
PRC-2 (MES-2/-3)	Epigenetic regulation H3K27methylation	EZH2, EED	[17] Patel et al., 2012
MES-4	Chromatin regulation	NSD proteins	[21] Gaydos et al., 2012
	H3K36 methylation		
FACT	Histone chaperone	SSRP1, SUPT16H	[28] Kolundzic et al., 2018
(HMG-3/-4, SPT-16)			
MRG-1	Part of NuA4 histone acteyltransferase complex	MRG-15	[35] Hadjuskova et al., 2019
RBBP-5	Set1/MLL methyltransferase complex member	RBBP5	[40] Kazmierck et al., 2020
